# Supplementary material for: Role of comprehensive geriatric assessment in healthcare of older people in UK care homes: realist review
Source: BMJ Open. 2019 Apr 8;9(4):e026921. doi: 10.1136/bmjopen-2018-026921 (PMC6500328; doi:10.1136/bmjopen-2018-026921)
Supplement: Supplementary file 1 [file bmjopen-2018-026921supp001.pdf]

	RAMESES checklist	Location in text and additional comments
1	**TITLE**	Includes key terms 'care homes' 'Comprehensive Geriatric Assessment' and 'realist review'
2	**ABSTRACT**	Programme theory is summarised. Also Strengths and Limitations of the method of the review are given
	**INTRODUCTION**	
3	Rationale for review	To describe the components of CGA, how they are applied within the care home setting and what outcomes have been reported
4	Objectives & focus	Introduction; Objectives of Realist Review
	**METHODS**	
5	Changes in the review process	Change in search terms; not using Geriatric Assessment MeSH term.<br>Presentations were given in care homes, but residents were not interviewed due to difficulty conveying putative theory of CGA
6	Rationale for realist synthesis	Introduction; Objectives of Realist Review
7	Scoping the literature	Methods; Stage 1. Stakeholder interviews, consultation with programme group of PEACH, PPI. Advised that scope should be CGA in care homes, therefore excluded Evercare community matron literature. Included InterRAI where this is used for care planning
8	Searching processes	Methods; Stage 1. Requested literature from programme group of PEACH, discussed on twitter meaning of CGA, spoke to senior professionals in OT & SLT. Searches focused on CGA in CH, with wider reading of MDT and care planning literature.
9	Selection and appraisal of documents	Methods; Stage 2. Relevance to rough programme theory. Preference for empirical data for outcomes. Articles whose scope was epidemiological study or study of one specific clinical topic were excluded
10	Data extraction	Methods; Stage2. Text of articles was coded according to relevance to 3 'nested' CMO configurations. Emphasis on configuration (not simply listing M's and O's) and relationship between CMOs
11	Synthesis processes	Methods; Stage 3. Focus on multidisciplinary perspectives, technologies that facilitate this, processes to ensure personalisation or person-centred
	**RESULTS**	
12	Document flow diagram	Flow schematic - Fig 1.
13	Document characteristics	Table of articles - Appendix 1. Includes description of type of study, quality and usefulness for this review, summary of outcomes relevant to this review
14	Main findings	Theory building - 3 CMO configurations. Difficulty in testing phase due to limited literature
	**DISCUSSION**	
15	Summary of findings	Description of programme theory in CMO configuration, giving contrast to published systematic reviews
16	Strengths, limitations and future research directions	Overall strength of evidence for description of theory of how CGA works in practice. Strength of comprehensive search of literature and extraction of data on context.
		Limitations; lack of evidence on the specific process of converting the assessment into the care plan
		Gaps identified for future research; lack of evidence of how communication between professionals occurs in practice, lack of evidence of how care plans are put into practice by care assistants, lack of evidence of how residents and family are involved in decision-making
17	Comparison with literature	Existing literature focuses on assessment tools but little about processes and teamwork; these are implicit rather than described
18	Conclusion and recommendations	We present a programme theory for CGA in care homes and recommend that this can be used to support quality improvement and future research
19	**Funding**	Dunhill Medical Trust
