# Supplementary material for: Role of comprehensive geriatric assessment in healthcare of older people in UK care homes: realist review
Source: BMJ Open. 2019 Apr 8;9(4):e026921. doi: 10.1136/bmjopen-2018-026921 (PMC6500328; doi:10.1136/bmjopen-2018-026921)
Supplement: Supplementary file 2 [file bmjopen-2018-026921supp002.pdf]

First author	Year	Ref #	Type of study	Setting	Location	Population	Assessment or Intervention	Quality of data extracted*
Adra	2017	24	Grounded theory	Nursing home	Lebanon	-	Unstructured	2
Andrew	2018	35	Observational before/after	Care home	Canada	-	LTC-CGA / Care by Design	2
Marshall	2015	12, 26, 35	Mixed methods	Care home	Canada	Average age 85	LTC-CGA / Care by Design	3
Bellantonio	2008	34	RCT	Assisted living	US	Dementia	Systematic multidisciplinary assessment	3
Harvey	2014	33	RCT	Residential home	Australia	65yrs & older	RECIPE / Postdischarge	3
Banerjee	2015	47	Qualitative review	Care home	Canada	-	MDS/RAI	1
Boorsma	2011	27	Cluster RCT	Residential home	Netherlands	75yrs & older	RAI-LTCF(web)	3
Challis	1999	40	Review	Care home	UK	-	MDS/RAI	1
Dellefield	2015	45	Review	Care home	-		Comprehensive care plan MDS/RAI	1
Holtkamp	2001	25	Non-randomised controlled study	Nursing home	Netherlands	Mean age 76 & 83	MDS/RAI	3
Kontos	2009	46	Qualitative	Nursing home	Canada	-	MDS/RAI	2
Thomas	2014	42	Retrospective cohort analysis	Nursing home	US	Average age 80	MDS3	3
Salva	2009	44	Review & consensus	Care home	-	-	MDS / Mini-Nutritional Assessment	1
Stewart	2003	36	Editorial	Care home	-	-	MDS/RAI	1
Burns	2011	39	Narrative review	Care home	-	-	Medication review	1
Bien	2005	43	Narrative review	Hospital, care home, community	-	-	-	1
Worden	2006	37	Observational	Care home	UK	-	-	2
Panza	2017	38	Systematic review	Care home	-	-	-	3
Meyer	2009	50	Editorial	-	-	-	-	1

First author	Year	Outcome 1	Outcome 2	Outcome 3
Adra	2017			
Andrew	2018	Polypharmacy (>10 meds) decreased from 86.8% to 79.5%		
Marshall	2015	Advanced directive 56.4%		Information transfer for emergency care significantly increased (P < .001)
Bellantonio	2008			Decreased unanticipated transitions (13%), hospitalisations (45%), emergency transfers (12%), death (63%)
Harvey	2014	Advanced directive increased from 13% to 67%	Decreased outpatient visits from 76% to 37%	Satisfaction increased from 58% to 95%
Banerjee	2015			
Boorsma	2011		40 MDTs were held	Quality of care mean difference -6.7 (p = 0.009) medium effect size 0.72
Challis	1999			
Dellefield	2015			
Holtkamp	2001	Number of identified needs increased in experimental; decreased in control		
Kontos	2009			
Thomas	2014			
Salva	2009			
Stewart	2003			
Burns	2011			
Bien	2005			
Worden	2006			
Panza	2017	Identified oral & dental needs		
Meyer	2009			
